# Antimicrobial Resistance and Causal Relationship: A Complex Approach Between Medicine and Dentistry

**DOI:** 10.3390/medicina61101870

**Published:** 2025-10-18

**Authors:** Giovanni Caivano, Fabio Massimo Sciarra, Pietro Messina, Enzo Maria Cumbo, Luigi Caradonna, Emanuele Di Vita, Salvatore Nigliaccio, Davide Alessio Fontana, Antonio Scardina, Giuseppe Alessandro Scardina

**Affiliations:** Department of Precision Medicine in Medical, Surgical and Critical Care (Me.Pre.C.C.), University of Palermo, Via Del Vespro, 129, 90127 Palermo, Italy; caivanolegale@tiscali.it (G.C.); fabiomassimosciarra@gmail.com (F.M.S.); pietro.messina01@unipa.it (P.M.); enzo.cumbo@unipa.it (E.M.C.); luigi.caradonna@unipa.it (L.C.); emanueledivita95@gmail.com (E.D.V.); salvo.nigliaccio@gmail.com (S.N.); davidealessiofontana@libero.it (D.A.F.); antoscardi213@gmail.com (A.S.)

**Keywords:** antimicrobial resistance, antibiotics, public health, infectious diseases, interdisciplinary approach

## Abstract

Antimicrobial resistance (AMR) is widely recognized as a major global public health threat, yet its origins and implications extend beyond the simple misuse or overuse of antibiotics. This study explores AMR as a complex, multifactorial phenomenon shaped by biological, clinical, dental, environmental, and social dynamics, with particular attention to the emerging role of dentistry. A narrative literature review was performed, drawing from textbooks, peer-reviewed articles, and official World Health Organization (WHO) reports, with emphasis on recent findings on periodontal biofilms as reservoirs of resistance genes. The analysis shows that AMR develops through bacterial mutations, horizontal gene transfer, environmental contamination, healthcare-associated practices, and patient behaviors, all of which interact to sustain its spread. Within dentistry, subgingival microresistances are gaining relevance, complicating treatment strategies and underscoring the need for more conscious clinical decision-making. The findings suggest that reducing antibiotic prescriptions or developing new drugs alone will not suffice; instead, a systemic, interdisciplinary approach is required, integrating microbiology, clinical practice, public health, and institutional responsibility. Such awareness is essential to confront the significant clinical, economic, and social implications of AMR and to foster strategies capable of addressing its complex and evolving nature.

## 1. Introduction

Antimicrobial resistance (AMR) is one of the most serious threats to global public health and has been identified by the World Health Organization (WHO) as one of the ten major global health emergencies [1,2]. AMR is not merely a therapeutic failure, nor a problem confined to the clinical setting, but a complex and systemic phenomenon rooted in biological mechanisms, environmental dynamics, medical practices, and social behaviors. Although these dimensions are acknowledged, they are not always clearly reflected in the available scientific literature [3,4,5].

Antimicrobial resistance cannot be regarded solely as a microbiological issue but constitutes an interdisciplinary challenge encompassing human–environment interactions, prescribing culture, the organization of healthcare institutions, and the very nature of scientific knowledge production [3,4,5,6,7,8,9,10].

In such a complex context, developing a scientific approach based on causal analysis and capable of navigating complexity is essential. Focusing only on coincidences or apparent correlations risks oversimplifying a global and multifactorial phenomenon into linear frameworks that fail to capture its full scope.

Integrating biological, environmental, clinical, social, and institutional factors allows for understanding antimicrobial resistance as a dynamic interplay of evolving causes that requires multidimensional responses [3,10].

Science, when facing complex phenomena, advances not through absolute truths but through observation, verification, error, and continuous hypothesis revision. Examining gaps and limits in prior knowledge fosters critical thinking, with reasoning as a central research tool. Some experiential approaches do not quantify data but guide directions and map pathways, often beyond the laboratory, through philosophical reflections on fundamental questions.

This study goes beyond identifying the causes of AMR to examine how these causes are conceived and addressed. In the medical–biological context, the notion of a “causal relationship” is not definitive evidence but a rational, provisional, and context-dependent construct.

The lack of immediate solutions should be seen not as a gap to fill with superficial explanations but as an opportunity to maintain an open field of inquiry and generate new questions. Critical reflection allows hypotheses to be assessed carefully, essential factors to be distinguished from secondary ones, and research directions to be identified even when results are unclear.

Accordingly, this work analyzes the causes of antimicrobial resistance from a broad interdisciplinary perspective, integrating biology, medicine, dentistry, ecology, and health policy, to enhance understanding of the phenomenon and support more effective intervention strategies.

## 2. Materials and Methods

For the preparation of this work, a narrative literature review was conducted with the aim of analyzing the main scientific evidence concerning antimicrobial resistance (AMR) and its implications in medical and dental practice. The review focused on identifying biological mechanisms, environmental determinants, and clinical behaviors contributing to the spread of AMR across interdisciplinary contexts, particularly emphasizing the interconnection between medicine and dentistry [2,3,6,10].

### 2.1. Search Strategy and Study Selection

The literature search was performed up to June 2024 across four major databases: PubMed, Scopus, ResearchGate, and Google Scholar.

The following combinations of keywords and Boolean operators were used: “antimicrobial resistance”, “antibiotic resistance”, “oral microbiome”, “dentistry”, “biofilm”, “antibiotic stewardship”, “horizontal gene transfer”, and “One Health” (using “AND”/“OR” to refine results).

Studies were included if they met the following criteria:

1. Published in English or Italian;
2. Addressed antimicrobial resistance in medicine, dentistry, or environmental microbiology;
3. Appeared in peer-reviewed journals or official institutional reports (e.g., World Health Organization, European Centre for Disease Prevention and Control) [2,11].

Exclusion criteria comprised: editorials, short communications, conference abstracts, case reports, non-peer-reviewed material, and articles lacking full text or methodological clarity.

In addition to journal articles, textbooks, and official guidelines from the World Health Organization (WHO) and related agencies were reviewed to contextualize surveillance data, clinical recommendations, and mitigation strategies [2,11].

### 2.2. Search Strategy and Study Selection

Two authors independently screened titles and abstracts to identify studies meeting the inclusion criteria. Full-text versions were then evaluated to extract information regarding:

- Molecular and genetic determinants of AMR;
- Environmental and behavioral factors influencing resistance selection;
- Clinical and therapeutic implications in medical and dental settings.

Discrepancies between reviewers were resolved through consensus meetings. The extracted data were organized into three thematic categories reflecting the multifactorial model of AMR:

(a) Molecular and genetic determinants.
(b) Environmental and behavioral factors.
(c) Clinical and institutional implications in medicine and dentistry.

This thematic synthesis allowed for the integration of evidence across disciplinary boundaries, consistent with the study’s aim to approach AMR as a complex, systemic phenomenon.

### 2.3. Methodological Limitations

As a narrative review, this work does not include quantitative meta-analysis or statistical pooling of results. Consequently, heterogeneity among studies and potential publication bias cannot be entirely excluded. However, this methodology enables a broad, interdisciplinary synthesis of current evidence and conceptual frameworks, aligning with the epistemological perspective of this paper—namely, the need to interpret AMR through interconnected biological, clinical, environmental, and social dimensions [3,10].

## 3. Discussion

1. The Causal Relationship: Between Science, Perception, and Responsibility.

AMR calls for reflection that goes beyond clinical practice or environmental dynamics, involving the ways in which causal relationships are constructed and interpreted in medicine. The causal link is not an objective or immediately observable reality, but a logical and argumentative construct based on evidence, probability, and inference. In daily practice, however, the probable is often treated as certain and the useful as absolute, fostering a form of “naïve realism” that has, over time, produced convictions later revealed as fragile.

History shows numerous cases in which causality was interpreted hastily or simplistically, with significant clinical and social consequences. Notable examples include the following:

- Plastic as a safe material: long considered harmless and stable, it is now studied for its toxic effects, including potential endocrine and inflammatory impacts.
- The dogma of internal organ sterility: the discovery of the microbiome revealed stable microbial communities with regulatory functions, overturning the idea of sterile organs.
- The myth of absolute screening efficacy: tools such as mammography or PSA testing, initially perceived as guarantees of reduced mortality, have shown limitations related to overdiagnosis and overtreatment.
- The paradigm of immutable DNA: the deterministic view of the genotype has been replaced by epigenetics, which highlights the environment’s influence on gene expression.
- Unlimited trust in antibiotics: excessive and indiscriminate use, supported by the drug–cure paradigm, has driven the development of resistance, now one of the main global health emergencies [5,10].

These examples demonstrate that the construction of causal links is never neutral and requires constant critical reassessment in light of emerging evidence.

In dentistry, this issue is particularly relevant: the dynamics of oral biofilm cannot be reduced to a simple cause–effect relationship between microorganism and disease, nor can the use of antibiotics be seen as a universal solution. A more nuanced understanding of causality allows clinicians to avoid oversimplifications and to make therapeutic decisions that are more informed, effective, and sustainable.

2. The Epistemological Lesson: Complexity and Prudence.

These examples do not undermine the value of science but rather call for critical reflection on its development. Many established scientific “truths” stem from simplified models of causal relationships and may be shaped by technological limitations, cognitive biases, political pressures, or economic interests. When causality is assumed yet difficult to verify, initial solutions can inadvertently generate new problems.

In dentistry, the routine prescription of antibiotics for prophylactic purposes during surgical procedures was traditionally justified by the expectation of reducing postoperative infections. Recent evidence, however, indicates that actual benefits are limited, while the risk of promoting antimicrobial resistance (AMR) is significant [7,12,13]. This example underscores the importance of distinguishing apparent correlations from true causality and adopting prudent, evidence-based strategies.

Addressing complex phenomena such as AMR requires flexible thinking capable of discerning correlations from causation, individual risks from systemic issues, and short-term remedies from sustainable strategies. Well-established frameworks can guide decision-making within complex contexts, supporting prudent choices even in the face of incomplete data. Applied to AMR, these approaches reveal that the phenomenon extends beyond microbiology, encompassing ecological, social, political, and economic dimensions. Effective management therefore demands a systemic and interdisciplinary approach that integrates clinical interventions, professional education, and public awareness initiatives.

3. Molecular and genetic determinants of AMR.

### 3.1. Bacterial Genetics and Molecular Mechanisms of Resistance

AMR originates from the remarkable genetic plasticity of microorganisms. Bacteria exhibit a high evolutionary capacity that allows rapid adaptation to environmental stresses, including exposure to antimicrobial agents [3,6]. This high evolutionary capacity is driven by the combined effects of selective pressure and genetic plasticity. Selective pressure arises from repeated exposure to antimicrobial agents and environmental stressors, which favor the survival of mutant or horizontally transformed bacteria with advantageous traits. Genetic plasticity, in turn, is sustained by mobile genetic elements such as *plasmids*, *transposons*, and *integrons*, which facilitate the acquisition and recombination of resistance determinants across species and ecological niches. Together, these mechanisms enable bacterial populations to adapt rapidly, diversify metabolically, and persist in hostile environments, including oral and hospital biofilms. The main mechanisms underlying resistance are described below.

Spontaneous Mutations

During DNA replication, random errors can occur. Some of these mutations alter the antibiotic target, such as key proteins or enzymes, rendering it unrecognizable to the drug without compromising bacterial viability. Although stochastic, these mutations are positively selected in environments with high antibiotic pressure [3,4,10,14].

Horizontal Gene Transfer (HGT)

In addition to mutations, bacteria possess an even more powerful evolutionary mechanism: horizontal gene transfer, through which genetic material can be exchanged between unrelated bacteria [3,4,15], as observed for instance in *Staphylococcus aureus* acquiring the *mecA* gene conferring methicillin resistance, or *Enterococcus faecalis* transferring vancomycin-resistance genes (*vanA*, *vanB*) to other Gram-positive species. This phenomenon occurs via several processes:

- Conjugation: the direct transfer of extrachromosomal DNA, typically plasmids carrying antibiotic resistance genes, from one bacterium to another via a cytoplasmic bridge known as a sex pilus. A well-known example is the plasmid pOXA-48, which mediates carbapenem resistance in *Enterobacteriaceae* and can spread rapidly across different bacterial genera.
- Transformation: incorporation of free DNA present in the environment, frequently released by dead bacteria.
- Transduction: gene transfer mediated by bacteriophages, viruses capable of accidentally transporting genetic material from one bacterial host to another.

These mechanisms enable the rapid dissemination of entire clusters of resistance genes, often organized into genetic cassettes or resistance islands, within diverse bacterial communities, including those present in humans, animals, and the environment.

Interaction with Environmental Pollution.

Particularly relevant is the phenomenon of co-selection, in which resistance genes confer protection not only against antibiotics but also against heavy metals such as mercury, arsenic, or cadmium, commonly present in environments contaminated by industrial or agricultural waste. For example, the *merA* and *czcA* genes, detected in *Streptococcus mitis* and *Pseudomonas aeruginosa*, confer tolerance to mercury and cadmium, respectively, while being co-located on mobile genetic elements that also harbor β-lactam or macrolide resistance determinants. This highlights how environmental pollution can act as a selective driver for multidrug-resistant bacteria, even in the absence of direct antibiotic exposure [Table 1].

The phenomenon highlights the interplay between ecology and microbiology: soil, water, air, and favorable environmental conditions act as evolutionary laboratories where resistance genes emerge and stabilize [3,16,17].

### 3.2. Biological Mechanisms of Resistance

Once bacteria acquire the genetic capacity to resist, they employ specific molecular strategies to evade or neutralize the effect of antibiotics. The main mechanisms include:

Production of Inactivating Enzymes.

Some bacteria synthesize enzymes capable of destroying or chemically modifying the antibiotic before it can exert its effect. The most well-known example is β-lactamases, enzymes that hydrolyze the β-lactam ring of penicillins and cephalosporins, rendering them ineffective. Other enzymes include aminoglycoside-modifying enzymes and chloramphenicol acetyltransferases [18,19].

Modification of Molecular Targets.

Bacteria can alter the structure of the drug target, such as ribosomal subunits, DNA gyrase, or cell wall transpeptidases, preventing antibiotic binding without impairing cellular function. These modifications often result from point mutations or genes acquired via horizontal gene transfer [20,21].

Reduced Permeability.

Some bacteria modify porins or other membrane channels, limiting antibiotic entry. This mechanism is particularly relevant in Gram-negative bacteria, which possess an additional outer membrane that acts as a supplementary barrier [22].

Active Efflux.

Many bacteria produce efflux pumps, such as the AcrAB-TolC system, which actively expel antibiotics from the cell. These pumps can be specific or multidrug (MDR), contributing to cross-resistance [23,24].

Biofilm Formation.

Biofilms are three-dimensional structures composed of microbial communities adherent to natural or artificial surfaces, embedded in a matrix of polysaccharides, proteins, and extracellular DNA. Within biofilms, bacteria benefit from physical protection against antibiotics, slowed metabolism that reduces susceptibility, and intercellular communication via quorum sensing, enhancing cooperation and virulence. Biofilms also facilitate horizontal gene transfer, accelerating the evolution of resistance [25,26].

Subgingival biofilms in patients with chronic periodontitis have been found to harbor resistance genes spanning six antibiotic classes, with a predominance of macrolides. These biofilms are typically composed of complex microbial consortia dominated by *Porphyromonas gingivalis*, *Tannerella forsythia*, *Treponema denticola*, *Fusobacterium nucleatum*, and *Prevotella intermedia*, which cooperate through metabolic and signaling networks that enhance both virulence and gene exchange. The coexistence of these species within dense biofilms provides an optimal environment for horizontal gene transfer, explaining the persistence of resistant infections even under intensive therapy. This explains the difficulty in eradicating many chronic oral infections, even with prolonged or combination therapies, and underscores the importance of studying micro-resistances to develop novel therapeutic strategies [27].

### 3.3. Clinical Prescriptive Practices

Inappropriate Antibiotic Use.

Improper use of antibiotics represents a major iatrogenic driver of resistance selection and dissemination. Antibiotics are frequently prescribed for viral infections, such as the common cold, influenza, or non-bacterial pharyngitis, where they are ineffective yet still exert selective pressure on the commensal microbiota.

Incorrect dosing further exacerbates the problem: subtherapeutic doses may fail to reach effective concentrations, allowing bacterial survival and adaptation, while excessive doses disrupt the normal flora and promote the selection of resistant strains.

Premature discontinuation of therapy, upon initial symptom improvement, eliminates susceptible bacteria but allows resistant populations to persist and repopulate the host. Conversely, unnecessarily prolonged therapy increases bacterial exposure to antibiotics, enhancing adaptive selection opportunities.

Patient non-adherence, including delayed or missed doses, generates fluctuating plasma concentrations that favor the emergence of partially resistant strains. Collectively, these practices substantially contribute to reduced antibiotic efficacy and chronic infection persistence [7,8,28,29,30,31,32,33].

Pharmacological Interactions and Unintended Synergies.

In hospital settings, co-administration of antibiotics with other drugs, sometimes via the same intravenous route, may further compromise antimicrobial efficacy. Such combinations can alter the chemical stability of the active compound, modify solution pH or solubility, and trigger inactivation reactions.

Certain drug classes also interfere at pharmacokinetic or pharmacodynamic levels: proton pump inhibitors or antacids may reduce intestinal absorption of tetracyclines and quinolones, whereas enzyme inducers, such as antiepileptics, accelerate hepatic metabolism of antibiotics, lowering systemic concentrations [34,35,36].

Some combinations can produce pharmacological antagonism, wherein one drug diminishes the effect of another, resulting in therapeutic failure despite correct administration. These interactions, often under-investigated or underestimated, may promote resistant strain selection, clinical deterioration, increased healthcare costs, and heightened epidemiological risk.

### 3.4. The Environment as a Reservoir and Incubator of Resistance

The environment acts both as a reservoir and a selective niche for resistance genes, effectively serving as an evolutionary laboratory. Antibiotics used in human and veterinary medicine are excreted and frequently reach wastewater treatment plants, which do not always fully remove them; residual compounds can therefore contaminate rivers, soils, and oceans.

These persistent drugs, such as fluoroquinolones and macrolides, have been detected in fish, mollusks, and crustaceans downstream of wastewater treatment plants, subsequently entering the human food chain through bioaccumulation and consumption of contaminated seafood [17], while sewage sludge applied as fertilizer disseminates residues and resistance genes into agricultural soils. Wastewater systems also constitute genetic hotspots, where conditions favor the selection and horizontal transfer of resistance genes among environmental bacteria.

Furthermore, extreme climatic events, such as heatwaves or floods, can amplify the dissemination of pathogenic microorganisms and increase antibiotic usage, creating a vicious cycle whereby more infections lead to greater antibiotic exposure, ultimately accelerating the emergence and spread of resistance.

### 3.5. Human Behavior and Social Dynamics

Human behaviors play a pivotal role in the dissemination of antibiotic resistance. Self-medication and overuse are common in many countries where antibiotics can be purchased without a prescription, thereby increasing inappropriate usage.

Limited health literacy further exacerbates the problem, as many individuals are unaware of correct administration practices and the consequences of misuse.

Misinformation fosters misconceptions about the effectiveness of antibiotics against viral infections or fever, leading to unnecessary demands for these drugs. Finally, global mobility—including medical tourism, international travel, and migration—facilitates the transfer and circulation of resistant strains across continents [36,37].

### 3.6. Healthcare System and Nosocomial Infections

Nosocomial infections, also referred to as healthcare-associated infections (HAIs), are defined by the World Health Organization as infections that develop 48 h or more after hospital admission and were neither present nor incubating at the time of entry [37]. They currently affect approximately 7–10% of hospitalized patients in high-income countries and up to 15% in low- and middle-income regions, according to ECDC and WHO surveillance data [37,38].

In dentistry, although the prevalence is lower, nosocomial-like infections can occur in clinical settings through contaminated instruments, aerosols, and surfaces, particularly during oral surgical or periodontal procedures. This underscores the need for strict infection control protocols and adherence to updated WHO and ECDC guidelines within both medical and dental practice.

In high-intensity care units, such as intensive care, geriatrics, oncology, and major surgery, frequent use of broad-spectrum antibiotics promotes the selection of multidrug-resistant strains, including *Klebsiella pneumoniae*, *Acinetobacter baumannii*, *Pseudomonas aeruginosa*, and *Staphylococcus aureus*.

The risk is increased by inadequate sanitation and hygiene: surfaces, surgical instruments, and medical devices such as catheters, ventilators, or prostheses can become microbiological reservoirs if not properly disinfected or sterilized, facilitating cross-transmission of pathogens.

Insufficient training of healthcare personnel further exacerbates the problem, as a lack of updates on international guidelines, such as those from the WHO or ECDC, may result in improper practices, ranging from incorrect hand hygiene to improper use of personal protective equipment or inadequate management of antimicrobial therapies [37,38,39].

Another critical factor is insufficient microbiological surveillance, particularly in long-term care facilities or rehabilitation centers, where the absence of active screening, routine cultures, and reporting prevents monitoring of resistant strain circulation [38].

The situation is further complicated by the lack of standardized nomenclature, which hinders harmonization of epidemiological data across facilities, and by inadequate or absent nosocomial infection registries, making it impossible to identify patterns, monitor resistance trends, and implement timely corrective measures [39].

### 3.7. Transmission Pathways of Antimicrobial Resistance

Antibiotic resistance is transmitted not only as a genetic trait of bacteria but also through biological and environmental vectors. The environment represents a significant reservoir: waters contaminated with pharmaceutical residues or feces, agricultural soils treated with sludge or wastewater, and bioaerosols in the air can all carry resistant genes. Animals constitute another source of transmission, particularly through contaminated meat, direct contact with intensive farming operations, or zoonotic strains such as MRSA ST398 [40]. Human-to-human transmission occurs via droplets, unwashed hands, medical instruments, or contaminated environmental surfaces—a phenomenon particularly relevant in hospitals due to the high density of vulnerable patients, the use of invasive therapies, and the elevated turnover [37,39,41]

### 3.8. Clinical, Economic, and Social Impacts

Antimicrobial resistance represents one of the most critical challenges for modern medicine, as infections caused by multi-resistant bacteria no longer respond to conventional antibiotics. This results in therapeutic failures that require the use of reserve drugs, which are often more toxic, costly, and less effective, leading to increased mortality and morbidity, particularly among vulnerable populations. It also threatens numerous modern medical practices, rendering surgical procedures, transplants, intensive care, and oncological treatments more vulnerable.

The economic impact is substantial: direct costs include second- or third-line antibiotics, prolonged hospital stays, patient isolation, and increased diagnostic monitoring, while indirect costs involve productivity losses, long-term disability, premature mortality, and slowed healthcare processes.

The social consequences are equally significant: there is a crisis of trust in the healthcare system, with patients and professionals confused by infections that are difficult to treat; routine procedures may again result in severe outcomes, generating fear and uncertainty; the presence of resistant bacteria in the food chain compromises the agri-food sector; finally, psychological pressure on healthcare workers, patient stigmatization, and the risk of healthcare exclusion contribute to increased social inequalities [42,43].

### 3.9. Mechanistic Interplay Between Medicine and Dentistry

Although antimicrobial resistance is often studied within separate medical or dental frameworks, growing evidence highlights a continuous bidirectional exchange of resistance determinants between these domains. Oral bacteria such as Streptococcus mitis, Actinomyces spp., and Prevotella intermedia have been shown to carry resistance genes—ermB, tetM, mefA—that are also found in respiratory, intestinal, and nosocomial pathogens [25,26,27].

This overlap suggests that oral biofilms act as ecological reservoirs capable of sustaining and transferring resistance genes through horizontal gene transfer mechanisms, particularly conjugation and transformation, under antibiotic selective pressure. In this sense, the oral cavity represents not only a therapeutic target but also a potential hub for cross-resistance dissemination across systemic compartments.

Furthermore, dental procedures involving aerosol generation or antibiotic prophylaxis may facilitate the movement of resistant strains between patients and healthcare environments, reinforcing the notion of a shared resistance ecosystem encompassing both medicine and dentistry [7,12,13,25].

Understanding these molecular and ecological links is crucial to designing integrated control strategies that transcend disciplinary boundaries and address AMR as a unified One Health phenomenon [3,6,10].

### 3.10. Integrative Prevention Strategies at the Medicine–Dentistry Interface

Given the mechanistic and ecological overlap described above, coordinated antimicrobial stewardship between the medical and dental fields is imperative. Both disciplines share responsibility for antibiotic exposure, environmental release, and the emergence of multidrug-resistant bacteria.

Evidence supports the effectiveness of cross-sectoral stewardship programs, including shared antibiotic-prescription audits, interdisciplinary training, and harmonized prophylactic guidelines [33,37,38]. In dentistry, adherence to evidence-based protocols—limiting prophylaxis to high-risk patients, optimizing dosage and duration, and monitoring subgingival resistance patterns—represents a concrete preventive measure [12,13].

At the institutional level, integration within the One Health framework should include joint monitoring of antibiotic consumption, surveillance of resistance genes in oral microbiota, and the implementation of infection control practices aligned with WHO and ECDC standards [2,37,38] [Table 2].

Such interdisciplinary coordination promotes not only clinical effectiveness but also environmental sustainability and public health protection, reducing the global burden of AMR through preventive synergy rather than isolated interventions [3,10,33].

## 4. Bacterial Resistance: An Example of How a Complex Phenomenon Operates

Antimicrobial resistance (AMR) goes beyond the technical-scientific aspect, as it reflects our relationship with knowledge, responsibility, and the future of healthcare. Resistance processes follow complex and often invisible trajectories, not always attributable to individual behaviors, and require a systemic approach integrating microbiology, ecology, pharmacology, economics, and culture.

Understanding AMR challenges the traditional paradigm that views microbes solely as enemies, promoting awareness that sterility does not necessarily equate to health, that the microbiome represents a dynamic balance between host and microorganisms, and that conventional judgments about the role of microbes need reconsideration.

From an epistemological and philosophical perspective, science requires guiding tools such as doubt, proportion, and awareness of its own limits. The philosophy of medicine stimulates critical thinking, encourages reflection on the meaning of care, and contributes to developing a humanistic vision of medicine.

From a microbiological standpoint, bacteria should be considered evolutionary entities, capable of adaptation, communication, and genetic exchange. Managing AMR therefore implies coexistence with a complex microbiome and recognition of the essential link between humans and their surrounding microbial environment.

At the political and institutional level, managing AMR cannot be limited to blaming individual professionals but must address the systemic roots of the phenomenon.

Health policies are needed to promote research, continuous professional education, the availability of clear and shared guidelines, and the development of a culture of responsibility.

Finally, on an ethical level, the causal link should guide investigation and care without becoming a tool of accusation. Conversely, threats, fear, or regulatory pressure risk distorting ethics, reducing clinical practice to mere automatism.

## 5. Conclusions

The fight against antimicrobial resistance cannot be won solely through new drugs or reduced prescriptions; it requires awareness and collaboration across knowledge, ethics, and institutions [44]. AMR demonstrates that its causes are not linear but intertwined in dynamic networks, and that deterministic thinking alone is insufficient.

This phenomenon teaches epistemic humility: seemingly certain truths can prove fragile, and science must engage in dialogue with philosophy to foster doubt, critical reflection, and understanding of invisible connections.

On an ethical level, responsibility becomes shared, involving physicians, patients, institutions, and industry. Bacterial resistance forces us to reconsider causality, knowledge, and accountability, restoring to science its proper role: not to predict everything, but to understand the world within its complex networks.

## Figures and Tables

**Table 1:** Examples of co-selection mechanisms involving antibiotic and metal resistance genes in bacteria relevant to medicine and dentistry.

Gene	Mechanism/Function	Target or Resistance Type	Associated Antibiotic Resistance	Bacterial Species (Examples)
*merA*	Mercuric reductase; detoxifies Hg^2+^	Mercury resistance	β-lactams, macrolides (co-localized genes)	*Streptococcus mitis*, *Pseudomonas aeruginosa*
*czcA*	Efflux pump for Cd^2+^, Zn^2+^, Co^2+^	Heavy metal efflux	Tetracyclines, quinolones	*Pseudomonas* spp., *Enterococcus faecalis*
*arsB*	Arsenite efflux transporter	Arsenic detoxification	Sulfonamides (co-selection)	*Streptococcus oralis*, *Escherichia coli*
*copA*	Cu^+^-translocating ATPase	Copper resistance	Aminoglycosides	*Enterococcus faecium*, *Actinomyces* spp.

**Table 2:** Comparative overview of antimicrobial resistance mechanisms and implications in medicine and dentistry.

Domain	Common Bacteria	Main Resistance Genes/Mechanisms	Clinical Impact	Preventive Strategies
Medicine	*Escherichia coli*, *Klebsiella pneumoniae*, *Staphylococcus aureus*, *Pseudomonas aeruginosa*	*blaCTX-M*, *mecA*, *ndm-1*, efflux pumps (AcrAB-TolC)	Hospital-acquired infections, treatment failure, prolonged hospital stay	Antimicrobial stewardship programs, infection-control policies, surveillance of hospital environments
Dentistry	*Porphyromonas gingivalis*, *Tannerella forsythia*, *Streptococcus mitis*, *Actinomyces* spp.	*ermB*, *tetM*, *mefA*, β-lactamase production	Periodontal and endodontic infections, reduced prophylactic efficacy, oral–systemic dissemination	Evidence-based antibiotic prophylaxis, oral biofilm control, adherence to WHO/ECDC guidelines
Shared Environment	Aquatic and soil microbiota; opportunistic human commensals	Integrons, transposons, conjugative plasmids, metal-resistance genes (*merA*, *czcA*)	Environmental dissemination, cross-sector transmission to humans and animals	Wastewater management, reduction of pharmaceutical residues, One Health-based monitoring

## Data Availability

No new data were created or analyzed in this study.

## Abbreviations

The following abbreviations are used in this manuscript:

AMR	Antimicrobial Resistance
WHO	World Health Organization
HGT	Horizontal Gene Transfer
MDR	Multidrug Resistance
PSA	Prostate-Specific Antigen
ECDC	European Centre for Disease Prevention and Control
ICU	Intensive Care Unit
